# Key Predictors of Adherence to a Mobile Health App for Managing Chronic Spontaneous Urticaria

**DOI:** 10.1002/clt2.70110

**Published:** 2025-11-15

**Authors:** Hugo Viegas, Bernardo Sousa‐Pinto, Rafael José Vieira, Aiste Ramanauskaite, Ellen Witte‐Händel, Ana Gimenez‐Arnau, Carole Guillet, Claudio Alberto Salvador Parisi, Constance Katelaris, Daria Fomina, Désirée Larenas‐Linnemann, Jorge Sánchez, Elizabeth Garcia, Hermenio Lima, Igor Kaidashev, Iman Nasr, Isabel Ogueta Canales, Ivan Cherrez‐Ojeda, Jean Bousquet, Jonathan A. Bernstein, Jonny Peter, Jose Ignacio Larco Sousa, Kanokvalai Kulthanan, Karsten Weller, Kiran Godse, Krzysztof Rutkowski, Lasma Lapina, Laurence Bouillet, Luis Felipe Ensina, Margarida Gonçalo, Maria Staevska, Mariam Ali Yousuf Al‐Nesf, Markus Magerl, Martin Metz, Martijn van Doorn, Mary Anne Castor, Maryam Khoshkhui, Michael Makris, Michihiro Hide, Mohamad Abuzakouk, Mona Al‐Ahmad, Murat Türk, Natasa Teovska Mitrevska, Niall Conlon, Nicole Nojarov, Pavel Kolkhir, Philip Li, Ramzy Mohammed Ali, Rand Arnaout, Riccardo Asero, Sabine Altrichter, Simon Francis Thomsen, Young‐Min Ye, Zenon Brzoza, Zuotao Zhao, Torsten Zuberbier, Frank Siebenhaar, Emek Kocatürk, Sophia Neisinger

**Affiliations:** ^1^ MEDCIDS—Department of Community Medicine Information and Health Decision Sciences Faculty of Medicine University of Porto Porto Portugal; ^2^ CINTESIS@RISE Centre for Health Technology and Services Research Health Research Network Faculty of Medicine University of Porto Porto Portugal; ^3^ Institute of Allergology Charité—Universitätsmedizin Berlin Corporate Member of Freie Universität Berlin and Humboldt‐Universität zu Berlin Berlin Germany; ^4^ Fraunhofer Institute for Translational Medicine and Pharmacology ITMP Immunology and Allergology Berlin Germany; ^5^ Department of Dermatology Urticaria Center of Reference, and Excellence (UCARE) Hospital del Mar IMIM Universitat Pompeu Fabra Barcelona Spain; ^6^ Department of Dermatology University Hospital Zurich Zurich Switzerland; ^7^ Adult and Pediatric Allergy Sections of the Italian Hospital of Buenos Aires Buenos Aires Argentina; ^8^ Department of Medicine Campbelltown Hospital and Western Sydney University Sydney New South Wales Australia; ^9^ Moscow City Research and Practical Center of Allergology and Immunology Clinical Hospital No 52 Moscow Healthcare Department Russia Angioedema/Urticaria Center of Reference and Excellence (ACARE, UCARE) Moscow Russia; ^10^ Department of Clinical Immunology and Allergology IM Sechenov First Moscow State Medical University (Sechenov University) Moscow Russia; ^11^ Department of Pulmonology Astana Medical University Astana Kazakhstan; ^12^ Center of Excellence in Asthma and Allergy Larenas Hospital Médica Sur Mexico City Mexico; ^13^ Group of Clinical and Experimental Allergy Hospital ‘Alma Mater de Antioquia’ University of Antioquia Medellín Colombia; ^14^ Faculty of Medicine Universidad de los Andes Allergy Section Fundación Santa Fe de Bogotá UNIMEQ ORL Bogotá Colombia; ^15^ LEADER Research Inc. and Division of Allergy and Clinic Immunology (UCARE) Medicine Department McMaster University Hamilton Ontario Canada; ^16^ Poltava State Medical University Poltava Ukraine; ^17^ Department of Clinical Immunology and Allergy Royal Hospital Muscat Oman; ^18^ Department of Dermatology Faculty of Medicine Pontifical Catholic University of Chile Santiago Chile; ^19^ University Dermatological Center (DermaCDU) Las Condes Chile; ^20^ Unit of Dermatology Rancagua Regional Hospital Rancagua Chile; ^21^ Universidad Espíritu Santo Respiralab Research Center Guayaquil Ecuador; ^22^ Division of Rheumatology Allergy and Immunology University of Cincinnati College of Medicine Cincinnati Ohio USA; ^23^ ACARE Centre Division of Allergy and Clinical Immunology Department of Medicine Groote Schuur Hospital University of Cape Town Lung Institute Cape Town South Africa; ^24^ Department of Allergy UCARE Clinica San Felipe Lima Peru; ^25^ Department of Dermatology Faculty of Medicine Siriraj Hospital Mahidol University Bangkok Thailand; ^26^ Department of Dermatology Dr D Y Patil Medical College and Hospital Navi Mumbai India; ^27^ Urticaria Clinic St John's Institute of Dermatology Guy's and St Thomas' Hospital London UK; ^28^ Allergic Diseases Diagnosis and Treatment Center Riga Stradins University Pauls Stradins Clinical University Hospital Riga Latvia; ^29^ French National Reference Center for Angioedema (CREAK) Internal Medicine Department Grenoble University Hospital La Tronche France; ^30^ Division of Allergy Clinical Immunology and Rheumatology Department of Pediatrics Federal University of Sao Paulo Sao Paulo Brazil; ^31^ Dermatology Faculty of Medicine University of Coimbra and University Hospital Coimbra Local Health Unit Coimbra Portugal; ^32^ Medical University of Sofia Sofia Bulgaria; ^33^ Allergy and Immunology Section Hamad General Hospital Hamad Medical Corporation Doha Qatar; ^34^ Department of Dermatology UCARE Erasmus MC Rotterdam the Netherlands; ^35^ Department of Pediatrics University of the Philippines Manila‐Philippine General Hospital Manila Philippines; ^36^ Allergy Research Center Mashhad University of Medical Sciences Mashhad Iran; ^37^ Allergy Unit 2nd Department of Dermatology and Venereology National and Kapodistrian University of Athens University General Hospital “Attikon” Athens Greece; ^38^ Department of Dermatology Hiroshima City Hiroshima Citizens Hospital Department of Dermatology Hiroshima University Hiroshima Japan; ^39^ Allergy & Immunology Department Respiratory Institute Cleveland Clinic Abu Dhabi Abu Dhabi UAE; ^40^ Faculty of Medicine Kuwait University Kuwait City Kuwait; ^41^ Division of Allergy and Clinical Immunology Erciyes University School of Medicine Kayseri Türkiye; ^42^ ReMedika General Hospital Skopje North Macedonia; ^43^ School of Medicine Trinity College Dublin St James's Hospital Dublin Dublin Ireland; ^44^ University of Hong Kong Hong Kong Hong Kong; ^45^ Department of Dermatology King Faisal Specialist Hospital & Research Center Riyadh Saudi Arabia; ^46^ Ambulatorio di Allergologia Clinica San Carlo Milan Italy; ^47^ Department of Dermatology and Venerology UCARE Kepler University Hospital Linz Austria; ^48^ Faculty of Medicine Center for Medical Research Johannes Kepler University Linz Austria; ^49^ Department of Biomedical Sciences Department of Dermatology Bispebjerg Hospital University of Copenhagen Copenhagen Denmark; ^50^ Department of Allergy and Clinical Immunology Ajou University School of Medicine Suwon Korea; ^51^ Division of Allergology Department of Internal Diseases Institute of Medical Sciences University of Opole Opole Poland; ^52^ Department of Dermatology and Venerology Beijing Key Laboratory of Molecular Diagnosis on Dermatoses National Clinical Research Center for Skin and Immune Diseases NMPA Key Laboratory for Quality Control and Evaluation of Cosmetics Peking University First Hospital Beijing China; ^53^ Department of Dermatology Bahçeşehir University School of Medicine Istanbul Türkiye

**Keywords:** adherence, chronic spontaneous urticaria, mHealth

## Abstract

**Background:**

Mobile health technologies may improve the management of chronic diseases, such as chronic spontaneous urticaria. However, effectiveness of mHealth tools largely depends on patient adherence, which can be influenced by various demographic, clinical, behavioural, psychosocial factors, and apps characteristics (appealing and simplicity of use). Understanding these adherence patterns is crucial for optimizing mHealth interventions. In this study, we aimed to assess adherence patterns associated to the use of CRUSE, a mHealth app designed for patients with CSU.

**Methods:**

We assessed users of the CRUSE app with self‐reported CSU or suggested by a physician. For each user, we evaluated the number of days they completed the CRUSE daily monitoring questionnaire (app adherence) within the first 3 months after installation. We constructed univariable and multivariable ordered beta regression models to identify predictors of 3‐month adherence to the app.

**Results:**

We analysed data from 2085 patients (66,114 days). Median adherence to the CRUSE app was of 22 days (24.4% of 90 days). In multivariable regression models, the variables more strongly associated with increased adherence to CRUSE included age (average increase = 0.16 percent points [pp] per additional year; 95% credible interval [CrI] = 0.08; 0.23 pp), male sex (average difference = 4.24 pp; 95% CrI = 1.77; 6.39 pp), being from a European country (average difference = 2.66 pp; 95% CrI = 0.59; 5.19 pp), and using monoclonal antibodies (average difference = 4.60 pp; 95% CrI = 2.26; 6.65 pp).

**Conclusions:**

Our findings suggest that age, male sex, residence in Europe, and the use of monoclonal antibodies are significant factors associated with increased adherence to the CRUSE app. These insights may help identify patient subgroups who would benefit most from mHealth support in managing CSU.

**Abbreviations**
AAS: angioedema activity score
AECT: angioedema control test
CSU: chronic spontaneous urticaria
DAG: Directed Acyclic Graph
GDPR: General Data Protection
mHealth: mobile health
PROMs: patient‐reported outcome measures
UAS: urticaria activity score
UCARE: urticaria centres of reference and excellence
UCT: urticaria control test

## 1 Introduction

Tackling chronic diseases is a pressing challenge for healthcare systems globally, which have historically been designed more centred around the management of acute conditions rather than around the provision of care needed for long‐term conditions. A growing number of calls have emphasized the necessity of reshaping healthcare systems and policies to focus on the proactive management of chronic diseases, especially in a patient‐centred way [1, 2, 3, 4]. Mobile health (mHealth) apps are increasingly available to help meet these needs, potentially offering substantial advantages for the monitoring of patients with chronic diseases. The potential of mHealth apps to improve patient care is such that a classification has been proposed to group their functionalities into those of (i) support of clinical diagnosis and/or decision‐making, (ii) improvement of clinical outcomes through behaviour change and enhancement of patient adherence and compliance, (iii) standalone digital therapeutics, and (iv) delivery of disease‐related education [5].

The usability of mHealth apps in monitoring chronic diseases has already been demonstrated in respiratory allergic conditions. Over 39,000 patients with rhinitis and/or asthma have utilized the MASK‐air app, contributing more than 600,000 days' worth of data [6]. The example of MASK‐air can potentially be expanded to other diseases, such as chronic spontaneous urticaria (CSU) whose patients would greatly benefit from a mHealth app. CSU is a prevalent and debilitating immune‐related condition with a relevant impact on the quality of life, work and school productivity, and leisure and social activities of affected patients [7, 8, 9, 10, 11, 12]. This impact along with the unpredictable nature of its signs and symptoms (i.e., wheals and angioedema) render the use of a mHealth app based on a patient diary particularly valuable for the monitoring of CSU. The CRUSE app, using validated patient‐reported outcome measures (PROMs), was developed to help the need for long‐term disease monitoring and ensure adequate care for patients with CSU. The CRUSE app was launched in March 2022 and has contributed to advancing some knowledge on CSU, including on characteristics and ‘real‐life’ behaviours of the patients with CSU, and on the properties of daily visual analogue scales for monitoring of CSU [13, 14]. Its PROMs offer the potential of personalised management and patient centred care but this is reliant on adherence to the use of the app [15]. In fact, as with medications, adherence to the apps can also be a concern (the similitudes can be demonstrated by the capacity of adapting a taxonomy for adherence to medications [16] into a taxonomy for adherence to mHealth apps—Table 1—to promote consistency, clarity, and the ability to quantify adherence‐related behaviours in the context of mHealth apps). Previous studies have not only pointed to the importance of direct and close contact between healthcare professionals and patients in the context of an integrated care involving mHealth [17], but also suggested that not all patients may be equally receptive to the possibility of using mHealth apps [18, 19]. In this context, it is relevant to assess whether there are characteristics of patients that may predict an increased usage of this kind of tool.

**TABLE 1:** Summary of the definitions for adherence applied to mHealth apps (adapted from [16]).

Taxonomy	Definition
Adherence to mHealth apps	The extent to which users engage with an mHealth app as intended, including consistent use, task completion, and sustained interaction over time.
	*Initiation* occurs when a patient begins to engage with the app, including downloading, registering, and completing initial setup or onboarding activities.
	*Discontinuation* occurs when a patient stops engaging with the app, either temporarily or permanently, with no further meaningful interaction.
	*Implementation* is the degree to which patients apply the app's features as intended, including correct, consistent, and timely execution of recommended actions.
Management of adherence	The process of monitoring and supporting patients' adherence to apps by health care systems, providers, patients, and their social networks.
Adherence‐related sciences	Disciplines that investigate the causes and consequences of discrepancies between initial use and sustained, consistent adherence to apps over time.

Therefore, our goal was to analyse usage patterns of CRUSE app in patients with CSU and to identify key predictors of adherence to the app. We aimed to understand how various factors influence 3‐month adherence to the CRUSE app after installation.

## 2 Methods

### 2.1 Study Design

We performed a study to identify factors that could predict higher adherence to the CRUSE app over 3 months. First, we employed univariable models to assess individual variables. Then, we developed a multivariable model to identify key predictors of usage, while accounting for potential confounding factors.

### 2.2 Setting and Participants

The CRUSE app was launched in March 2022 by the global network of Urticaria Centers of Reference and Excellence (UCARE) [20] and is now available in 22 languages and in 35 countries. It is freely available in Google Play and Apple App Stores. A patient organization from Germany was involved in the development, and the beta version was tested in March 2022 by patients in the outpatient clinic of the UCARE center Berlin.

We included daily monitoring data provided between July 2022 and June 2024 by CRUSE users who had installed the app for at least 3 months. CRUSE users include those who had installed the app themselves or upon clinician's suggestions (i.e., there was no formal recruitment to use the app). We included participants aged between 18 and 90 years and who reported in the app having CSU. For each included user, we considered the first 3 months (90 days) following the installation of the CRUSE app. We included only users who used the CRUSE app for at least 9 days (10% of the 90‐day period) to exclude tester users of the app and individuals who downloaded the app without having urticaria or angioedema.

### 2.3 Ethics

The anonymized data analysis from the CRUSE app received approval from the Charité—Universitätsmedizin Berlin ethics committee (EA4/184/23). Additionally, users consent to the app's terms of use and data fair use agreement during registration, permitting the anonymous use of their data for research purposes. CRUSE data are securely stored in CloudVPS in the Netherlands, adhering to ISO 27001 and ISO 13485 standards and complying with Regulation (EU) 2016/679 on General Data Protection (GDPR).

### 2.4 Data Sources and Participants

The CRUSE app comprises a daily monitoring questionnaire for registering the daily signs and symptoms of CSU and angioedema. The daily monitoring questionnaire includes the urticaria activity score (UAS) and the angioedema activity score (AAS) [21]. When patients open the daily monitoring questionnaire, their responses to the PROMs are only saved after all questions have been answered.

When completing the CRUSE daily monitoring questionnaire, users are also requested to provide their daily medication for CSU using a search function or a scroll list customised for each country and containing the most common prescribed and over‐the‐counter medications for CSU.

In addition to the daily monitoring questionnaire, CRUSE users are requested monthly to complete the Urticaria Control Test (UCT) and, if self‐reporting angioedema, the Angioedema Control Test (AECT) [21].

To assess predictors of CRUSE app adherence, we retrieved information on patients' age, sex, region (Europe vs. outside Europe), presence, frequency and time until disappearance of wheals and of angioedema, baseline UCT score, baseline AECT score, years since urticaria diagnosis, use of medication (H1‐antihistamines, corticosteroids and monoclonal antibodies), and existence and number of triggers. Table 2 provides additional information of some of the variables retrieved.

**TABLE 2:** Variables with multiple categories and continuous variables with specific items.

Variable	Type of variable	Description
Frequency of appearance of wheals^a^	Categorical	Comprises seven categories: — Never — Less than once per month — Once per month — Once per week — More than once per week — Almost every day — Every day
Time until wheals disappear^a^	Categorical	Comprises seven categories: — Less than 1 h — 1–6 h — 6–12 h — 12–24 h — 24–48 h — 48–72 h — More than 72 h
Urticaria control test (UCT) score	Continuous	A score of 16 indicates complete disease control. A score of < 12 indicates poorly controlled disease, and a score ≥ 12 identifies patients with well‐controlled CSU
Angioedema control test (AECT) score	Continuous	A score of < 10 indicates poorly controlled angioedema, a score ≥ 10 identifies patients with well controlled angioedema, and a score = 16 identify patients with complete control of angioedema

Abbreviation: CSU, Chronic spontaneous urticaria.

^a^ There are similar variables for angioedema: Frequency of appearance of angioedema, Time until angioedema to disappear.

### 2.5 Data Analysis

**FIGURE 1:**
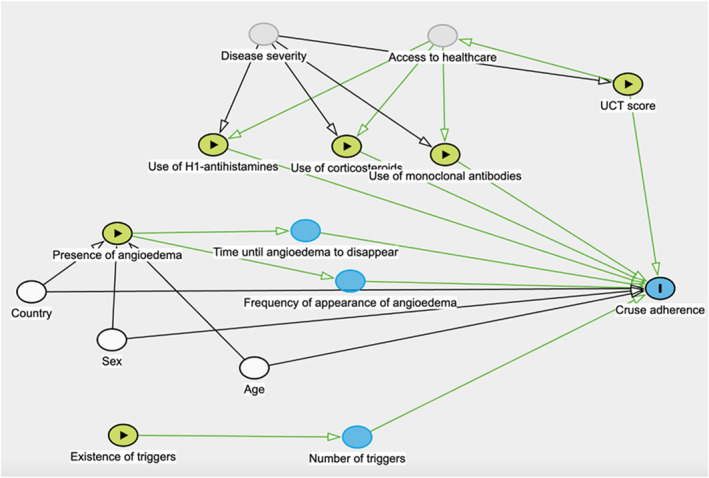
Directed acyclic graph (DAG) to select the independent variables to include in multivariable models.

Our outcome variable was adherence to the CRUSE app, defined as app usage percentage for the first 90 days after installation. As such, we developed univariable ordered beta regression models, including as independent variables each demographic and clinical variable for which information was retrieved [22]. Subsequently, we built a multivariable ordered beta regression model. To select the independent variables to include, we designed a Directed Acyclic Graph (DAG) (Figure 1). Included independent variables were presence of wheals and of angioedema, baseline UCT score, existence of triggers, use of medication (H1‐antihistamines, corticosteroids and monoclonal antibodies), age, sex and region.

For categorical variables, we interpreted the coefficients of the univariable ordered beta regression models as the average difference in app adherence (in percent points) between one specific category and the corresponding reference category. For continuous variables, the coefficients represent the average difference in adherence for every increase in one unit in the independent variable [22]. The logistic regression models' coefficients were interpreted as odds ratios (OR).

To assess the robustness of our findings, we performed a sensitivity analysis by defining the outcome dichotomously: having an adherence of an average of four or more days of CRUSE app usage per week. For this sensitivity analysis, we built univariable logistic regression models using the same independent variables as for the main analysis.

All analyses were performed using software R (version 4.3). For ordered beta regression models, we used the ordbetareg package in R to model our data. This package implements Bayesian models, with the priors of models' coefficients having been set to [mean = 0, standard‐deviation = 5], which are weakly informative for our data. Each model was run with at least 500 iterations and a single Markov chain. Convergence was assessed using the R‐hat statistic, with values greater than 1.1 indicating non‐convergence.

## 3 Results

We analysed data from 2085 patients, reporting a total of 66,114 days. Most days were reported by females (*N* = 46,584; 70.5%), and participants' mean age was 41.0 years (standard‐deviation = 13.7). Patients from European countries constituted 70.3% (*N* = 1465). Median adherence (app usage percentage for the first 90 days after installation) of CRUSE app was 24.4% (IQR = 33.3%) which corresponds to a median of 22 days (IQR = 30 days). Patients with adherence to CRUSE of 4 or more days per week corresponded to 19.8% (*N* = 413) of the total sample (Table 3).

**TABLE 3:** Demographics of CRUSE users and adherence to the app.

Variable	Descriptive statistics
*N* days [*N* users]	66,114 [2085]
Age—mean (SD)	41.0 (13.7)
Sex—*N* days (%)	
Males	19,232 (29.1)^a^
Females	46,584 (70.5)^a^
Other/unknown	298 (0.5)
European country—*n* (%)	1465 (70.3)
Adherence to the CRUSE app	
Median % of 90 days (IQR)	24.4 (33.3)
Median days (IQR)	22 (30)
Adherence to the CRUSE app 4 or more days per week	
*N* users (%)	413 (19.8)^b^

Abbreviations: IQR, Interquartile range; SD, Standard‐deviation.

^a^ *N* male users = 544 (26.1%), *N* female users = 1526 (73.2%).

^b^ *N* female users = 277 (67.1%).

### 3.1 Univariable Regression Models

In univariable regression models, increasing age and male gender was associated with an increased adherence to the CRUSE app (average difference of 0.16% points [pp] per year; 95% CrI = 0.10; 0.22 pp and average difference of 4.38 pp; 95% CrI = 2.22; 6.50 pp, respectively). Participants from European countries had an increased adherence to the app compared to the remainder (average difference of 2.57 pp; 95% CrI = 0.41; 4.87 pp). The use of medication, in particular monoclonal antibodies, was also associated with increased adherence to the app (average difference of 4.97 pp [95% CrI = 3.01; 6.97] (Table 4).

**TABLE 4:** Results of univariable models describing the association between each tested variable and 3‐month adherence to the CRUSE app.

Variable	Coefficient (95% credibility interval)
Age	0.16 (0.10; 0.22)
Male gender	4.38 (2.22; 6.50)
European country	2.57 (0.41; 4.87)
Presence of wheals	1.83 (−2.49; 5.34)
Frequency of appearance of wheals^a^	
Less than once per month	−3.67 (−15.40; 4.50)
Once per month	−2.77 (−14.90; 5.71)
Once per week	−7.41 (−19.10; 0.95)
More than once per week	−6.00 (−17.50; 1.78)
Almost every day	−4.74 (−14.40; 2.41)
Every day	−3.64 (−14.50; 3.52)
Time until wheals disappear^b^	
< 1 h	3.06 (−1.13; 7.38)
1–6 h	1.12 (−2.41; 5.29)
6–12 h	4.99 (1.19; 10.15)
12–24 h	5.00 (1.35; 9.36)
24–48 h	5.12 (0.89; 9.50)
48–72 h	4.45 (−1.23; 10.02)
UCT score	−0.03 (−0.25; 0.17)
Physical symptoms	0.38 (−0.69; 1.35)
Quality of life	−0.02 (−1.00; 0.81)
Insufficient treatment	−0.38 (−1.10; 0.36)
Overall control	−0.24 (−1.07; 0.52)
Presence of angioedema	1.50 (−0.83; 3.69)
Frequency of appearance of angioedema^a^	
Less than once per month	2.40 (−4.71; 12.00)
Once per month	5.22 (−2.28; 14.40)
Once per week	3.31 (−4.39; 12.10)
More than once per week	1.89 (−5.47; 11.80)
Almost every day	2.94 (−4.52; 11.70)
Every day	0.79 (−6.76; 11.40)
Time until resolution of angioedema^b^	
< 1 h	4.24 (−3.19; 11.92)
1–6 h	5.24 (−2.15; 11.59)
6–12 h	6.31 (0.13; 12.95)
12–24 h	7.18 (−0.04; 12.76)
24–48 h	6.74 (−0.75; 12.37)
48–72 h	2.51 (−4.82; 8.73)
AECT score	0.07 (−0.21; 0.34)
Frequency	0.45 (−0.67; 1.44)
Quality of life	0.62 (−0.64; 1.69)
Unpredictability	0.19 (−0.88; 1.29)
Treatment control	−0.34 (−1.18; 0.72)
Years since diagnosis	0.03 (−0.06; 0.16)
Existence of triggers	0.71 (−1.47; 3.16)
Number of triggers	0.30 (−0.39; 0.80)
Medication use	
Use of corticosteroids	0.67 (−1.72; 2.91)
Use of H1‐antihistamines	0.33 (−4.9; 6.15)
Use of monoclonal antibodies	4.97 (3.01; 6.97)

Abbreviations: AECT, Angioedema Control Test; UCT, Urticaria Control Test.

^a^ Reference category: Never.

^b^ Reference category: More than 72 h.

In our sensitivity analysis, where adherence to the app was dichotomized (defined as using the CRUSE app for an average of four or more days per week), we observed similar positive associations between adherence and increasing age (OR = 1.01; 95% CI = 1.01; 1.02), male gender (OR = 1.49; 95% CI = 1.18; 1.88), being from an European country (OR = 1.32; 95% CI = 1.04; 1.70) and use of monoclonal antibodies (OR = 1.64; 95% CI = 1.31; 2.06) (Table 5).

**TABLE 5:** Results of univariable logistic regression models describing the association between each tested variable and four or more mean days per week of adherence to the Cruse app during a 3‐month period.

Variable	OR (95% confidence interval)
Age	1.01 (1.01; 1.02)
Male gender	1.49 (1.18; 1.88)
European country	1.32 (1.04; 1.70)
Presence of wheals	1.06 (0.67; 1.72)
Frequency of appearance of wheals^a^	
Less than once per month	0.53 (0.22; 1.31)
Once per month	0.50 (0.21; 1.23)
Once per week	0.45 (0.18; 1.11)
More than once per week	0.38 (0.17; 0.87)
Almost every day	0.50 (0.24; 1.11)
Every day	0.47 (0.23; 1.02)
Time until wheals disappear^b^	
1–6 h	0.77 (0.18; 0.33)
6–12 h	1.01 (0.67; 1.51)
12–24 h	1.16 (0.80; 1.69)
24–48 h	1.34 (0.89; 2.01)
48–72 h	1.20 (0.68; 2.08)
> 72 h	0.75 (0.42; 1.31)
UCT score	1.01 (0.98; 1.04)
Physical symptoms	1.08 (0.98; 1.18)
Quality of life	1.04 (0.95; 1.14)
Insufficient treatment	0.97 (0.90; 1.06)
Overall control	1.03 (0.94; 1.13)
Presence of angioedema	1.04 (0.84; 1.30)
Frequency of appearance of angioedema^a^	
Less than once per month	0.95 (0.39; 2.70)
Once per month	1.26 (0.52; 3.56)
Once per week	0.85 (0.34; 2.45)
More than once per week	0.95 (0.38; 2.70)
Almost every day	1.09 (0.43; 3.13)
Every day	0.88 (0.33; 2.63)
Time until resolution of angioedema^b^	
1–6 h	0.90 (0.47; 1.83)
6–12 h	0.85 (0.44; 1.74)
12–24 h	0.96 (0.50; 1.93)
24–48 h	0.93 (0.48; 1.89)
48–72 h	0.78 (0.36; 1.72)
> 72 h	0.44 (0.15; 1.14)
AECT score	1.01 (0.98; 1.05)
Frequency	1.06 (0.94; 1.20)
Quality of life	1.10 (0.98; 1.24)
Unpredictability	1.03 (0.93; 1.15)
Treatment control	0.96 (0.86; 1.07)
Years since diagnosis	1.01 (0.99; 1.02)
Existence of triggers	1.15 (0.92; 1.45)
Number of triggers	1.03 (0.97; 1.10)
Medication use	
Use of corticosteroids	1.05 (0.81; 1.37)
Use of H1‐antihistamines	0.94 (0.52; 1.81)
Use of monoclonal antibodies	1.64 (1.31; 2.06)

Abbreviations: AECT, Angioedema Control Test; OR, Odds ratio; UCT, Urticaria Control Test.

^a^ Reference category: Never.

^b^ Reference category: More than 72 h.

### 3.2 Multivariable Model Describing the Association Between Relevant Variables and 3‐Month Adherence to the CRUSE App

Age (average difference = 0.16 pp per year; 95% CrI = 0.08; 0.23 pp), male gender (average difference = 4.24 pp; 95% CrI = 1.77; 6.39 pp) and being from a European country (average difference = 2.66 pp; 95% CrI = 0.59; 5.19 pp) were all associated in multivariable models with an increased adherence to the CRUSE app. As for medication, we found a positive adjusted association between the use of monoclonal antibodies and adherence to the CRUSE app (average difference = 4.60 pp; 95% CrI = 2.26; 6.65 pp) (Table 6).

**TABLE 6:** Results of the multivariable model describing the association between relevant variables and 3‐month adherence to the CRUSE app, adjusted for sex, gender and country.

Variable	Coefficient (95% credibility interval)
Age	0.16 (0.08; 0.23)
Male gender	4.24 (1.77; 6.39)
European country	2.66 (0.59; 5.19)
Presence of wheals	2.84 (−1.58; 7.10)
UCT score	−0.06 (−0.28; 0.15)
Presence of angioedema	1.84 (−0.30; 3.89)
Existence of triggers	1.23 (−0.84; 3.03)
Medication use	
Use of corticosteroids	0.19 (−2.23; 2.38)
Use of H1‐antihistamines	1.16 (−5.38; 7.00)
Use of monoclonal antibodies	4.60 (2.26; 6.65)

Abbreviation: UCT, Urticaria Control Test.

## 4 Discussion

In this study, we identified key predictors of adherence to the CRUSE app. Adherence was associated with demographic, geographic, and clinical factors including age, male gender, being from a European country, and the use of medication, particularly monoclonal antibodies.

The findings of this study align with existing literature on mHealth app usage while providing some unique insights into predictors of adherence. Consistent with a previous mHealth MASK‐air study [23], adherence to CRUSE was associated with treatment intensity, with patients on advanced therapies such as monoclonal antibodies demonstrating higher adherence. This may be attributed to these patients having more regular clinical follow‐up with specialized physicians (e.g., working at specialized UCARE centers) being more prone to promote app use. On the other hand, these results suggest an indirect association between disease severity and app adherence, as patients with more severe disease are more likely to use medication, particularly monoclonal antibodies. This contrasts with the negative findings observed with variables more directly related to CSU severity or presentation. However, these patients' symptoms in these patients may have been under control due to biological treatment.

The positive association between age and adherence contrasts with typical trends in digital health, where older individuals often exhibit lower engagement due to unfamiliar technology and poor health literacy [24]. However, in this study, the mean age of users was 40 years, suggesting a middle‐aged adult cohort. Therefore, this effect may have been mitigated, as app usability and health motivations likely outweighed age‐related technological challenges in this group. In addition, even though users of higher age groups may display higher adherence (Figure S1), there is a lower number of participants (and, therefore, of observations) from such users. This suggests that, in relation to older patients, clinicians may have carefully selectively recommended the CRUSE app to those who were more likely to engage with it, potentially introducing a selection bias. Being from Europe was associated with increased adherence, which could be explained by the fact that the CRUSE app was developed in Europe with physicians who were more involved in its development likely promoting its regular use. This emphasizes the importance of targeted efforts to broaden accessibility and usage across diverse demographics. Finally, male gender was significantly associated with adherence to the CRUSE app, in line with previous research which found that men had more intention to use mHealth apps than women [25, 26]. This finding could be explained by men being more open to use technology and seeking health‐related answers [27]. Also, differences in health behaviour, or the design and marketing of apps that might appeal more to male users.

This study has some limitations. Firstly, there is a selection bias as there may be an overrepresentation of patients who have better access to healthcare. Due to privacy concerns, we are not able to distinguish patients who downloaded the app by themselves or after being indicated by their physicians. Nevertheless, the positive association between medication use and adherence to the app suggests that patients with regular clinical follow‐up are more likely to use the app, possibly due to physician encouragement. This could be explored in a future study in UCARE centres. This also provides better control over the treatment regimen, which is particularly important for patients on cost‐intensive (i.e., total cost of care) treatments such as monoclonal antibodies. Moreover, as CRUSE app was developed in Germany, a significant proportion of users are from this country, which may limit the generalizability of findings to other regions. Reliance on self‐reported data introduces potential information bias, as it is unclear whether users consistently and accurately report their symptoms and medication use. Other biases should be considered. For some patients, CSU was self‐reported and not necessarily confirmed by a physician. Patients might stop using the app over time, especially if their symptoms improve, if CSU spontaneously remits over time, if they lose interest, or due to other medical conditions. This could result in a biased sample, as individuals who persist in using the app might experience a different disease burden compared to those who stop. Furthermore, the days on which patients engage with the CRUSE app might differ systematically from other days; for instance, patients may be more likely to use the app when their symptoms are more severe [13]. While these variables can in part explain adherence to the app, we did not measure them as we only considered factors related to patient baseline characteristics. A future focus group study involving users of the app might help answering some of these questions.

The current study has many strengths, including its assessment of a large sample, the evaluation of patients from multiple countries, and the relevance of the assessed question. Another strength concerns the models used to identify predictors of adherence and the fact that the candidate variables were selected after creating a DAG. The findings of this study suggest that, while mHealth apps like CRUSE can complement traditional care, their success depends on tailoring them to specific patient characteristics and contexts. In CSU, there is currently little evidence of reliable indicators that precede an exacerbation or flare‐up. However, by integrating mHealth apps into clinical practice, it may become possible to detect early changes and eventually identify predictive patterns—similar to asthma, where increased short‐acting beta agonists or worsening symptomatology often precede exacerbations [28]. Such insights would enable physicians and patients to discuss timely adjustments to treatment plans, thereby optimizing medication use through a blended‐care approach based on real‐time patient‐reported data [29, 30]. The embedding of artificial intelligence within CRUSE would open new possibilities for quantification of the impact of CSU and prediction of its course, facilitating clinicians to stratify patients by urgency of treatment needs [13, 31]. However, maximising the underlying gains requires understanding the acceptance of this kind of mHealth tool across diverse patient populations, and the implementation of strategies of improving its use among less adherent groups. Importantly, the design of such apps should be informed by clinical guidelines while remaining flexible enough to integrate the perspectives of both clinicians and patients, thereby enabling meaningful tailoring of care. Such would be essential to move from a one‐size‐fits‐all approach to a more personalized patient‐centred practice. Longitudinal studies are essential to evaluate the long‐term impact of app adherence on clinical outcomes and disease management.

In conclusion, we identified key predictors of adherence to the CRUSE app, highlighting specific subgroups more likely to engage with this tool for managing their CSU. These predictors include older age, male sex, residence in a European country and the use of certain medications, which may reflect higher disease severity and improved access to healthcare. This study underscores the potential of CRUSE to provide personalized care in CSU patients. In addition, it offers valuable insights into the factors influencing adherence to mHealth tools, with the methodology potentially applicable to other disease, paving the way for broader implementation of such tools in healthcare.

## Author Contributions

**Hugo Viegas:** conceptualization, data curation, formal analysis, visualization, writing – original draft, methodology, investigation, supervision, project administration, writing – review and editing, resources, validation, software. **Bernardo Sousa‐Pinto:** conceptualization, data curation, formal analysis, visualization, writing – original draft, methodology, investigation, supervision, project administration, writing – review and editing, software, validation, resources. **Rafael José Vieira:** conceptualization, data curation, formal analysis, visualization, writing – original draft, methodology, investigation, supervision, project administration, software, validation, resources. **Aiste Ramanauskaite:** writing – review and editing. **Ellen Witte‐Händel:** writing – review and editing. **Ana Gimenez‐Arnau:** writing – review and editing. **Carole Guillet:** writing – review and editing. **Claudio Alberto Salvador Parisi:** writing – review and editing. **Constance Katelaris:** writing – review and editing. **Daria Fomina:** writing – review and editing. **Désirée Larenas‐Linnemann:** writing – review and editing. **Jorge Sanchez:** writing – review and editing. **Elizabeth Garcia:** writing – review and editing. **Hermenio Lima:** writing – review and editing. **Igor Kaidashev:** writing – review and editing. **Iman Nasr:** writing – review and editing. **Isabel Ogueta Canales:** writing – review and editing. **Ivan Cherrez‐Ojeda:** writing – review and editing. **Jean Bousquet:** writing – review and editing. **Jonathan A. Bernstein:** writing – review and editing. **Jonny Peter:** writing – review and editing. **Jose Ignacio Larco Sousa:** writing – review and editing. **Kanokvalai Kulthanan:** writing – review and editing. **Karsten Weller:** writing – review and editing. **Kiran Godse:** writing – review and editing. **Krzysztof Rutkowski:** writing – review and editing. **Lasma Lapina:** writing – review and editing. **Laurence Bouillet:** writing – review and editing. **Luis Felipe Ensina:** writing – review and editing. **Margarida Gonçalo:** writing – review and editing. **Maria Staevska:** writing – review and editing. **Mariam Ali Yousuf Al‐Nesf:** writing – review and editing. **Markus Magerl:** writing – review and editing. **Martin Metz:** writing – review and editing. **Martijn van Doorn:** writing – review and editing. **Mary Anne Castor:** writing – review and editing. **Maryam Khoshkhui:** writing – review and editing. **Michael Makris:** writing – review and editing. **Michihiro Hide:** writing – review and editing. **Mohamad Abuzakouk:** writing – review and editing. **Mona Al‐Ahmad:** writing – review and editing. **Murat Türk:** writing – review and editing. **Natasa Teovska Mitrevska:** writing – review and editing. **Niall Conlon:** writing – review and editing. **Nicole Nojarov:** writing – review and editing. **Pavel Kolkhir:** writing – review and editing. **Philip Li:** writing – review and editing. **Ramzy Mohammed Ali:** writing – review and editing. **Rand Arnaout:** writing – review and editing. **Riccardo Asero:** writing – review and editing. **Sabine Altrichter:** writing – review and editing. **Simon Francis Thomsen:** writing – review and editing. **Young‐Min Ye:** writing – review and editing. **Zenon Brzoza:** writing – review and editing. **Zuotao Zhao:** writing – review and editing. **Torsten Zuberbier:** writing – review and editing. **Frank Siebenhaar:** writing – review and editing. **Emek Kocatürk:** conceptualization, writing – original draft, methodology, investigation, supervision, project administration, writing – review and editing, resources, funding acquisition. **Sophia Neisinger:** conceptualization, methodology, investigation, supervision, project administration, writing – review and editing, funding acquisition, resources.

## Conflicts of Interest

Hugo Viegas has no conflict of interest to declare in relation to this work.

Bernardo Sousa‐Pinto has no conflict of interest to declare in relation to this work.

Rafael José Vieira has no conflict of interest to declare in relation to this work.

Aiste Ramanauskaite has no conflict of interest to declare in relation to this work.

Ellen Witte‐Händel has no conflict of interest to declare in relation to this work.

Ana Gimenez‐Arnau has received consulting fees from Almirall, Amgen, Blue ‐Print, CELLDEX, ESCIENT, FAES, Genentech, GSK, Jaspers, Leo Pharma, Mitsubishi Tanabe, Novartis, Noucor, Sanofi–Regeneron, Thermo Fisher Scientific, Septerna, Servier, Uriach Pharma, grants or contracts with ESCIENT, NOUCOR, Novartis, Instituto Carlos III‐ FEDER, Uriach Pharma and Payments or honoraria for lectures from Almirall, Avene, Genentech, GSK, LEO‐PHARMA, Menarini, MSD, NOUCOR, Novartis, Sanofi, Uriach Pharma.

Carole Guillet has no conflict of interest to declare in relation to this work.

Claudio Alberto Parisi has no conflict of interest to declare in relation to this work.

Constance Katelaris has no conflict of interest to declare in relation to this work.

Daria Fomina has no conflict of interest to declare in relation to this work.

Désirée Larenas‐Linnemann Fomina has no conflict of interest to declare in relation to this work. Outside she declares payment to her centre for lectures from AZ, GSK, Sanofi, Novartis, and Chiesi.

Jorge Sánchez Fomina has no conflict of interest to declare in relation to this work.

Elizabeth Garcia has no conflict of interest to declare in relation to this work.

Hermenio Lima reports consultancy fees from AbbVie (Abbott), Amgen, AstraZeneca, Bristol‐Myers Squibb, Celgene, Dermira, Eli Lilly, Janssen, La Roche‐Posay, Merck Sharp & Dohme, Novartis, Pfizer, Regeneron, and Sanofi; personal payment. HL reports grants/grants pending for clinical trials from AbbVie (Abbott), Amgen, AstraZeneca, Bristol‐Myers Squibb, Celgene, Dermira, Eli Lilly, GSK, Incyte, Janssen, La Roche‐Posay, Merck Sharp & Dohme, Moonlake, Novartis, Pfizer, Regeneron, and Sanofi; personal payment. HL reports payment for lectures from AbbVie, Novartis, Sanofi, and Bausch Health; personal payment. HL reports payment for development of educational presentations from AbbVie (Abbott), Celgene, Janssen, Leo Pharmaceutics, Novartis, Sanofi, Pfizer, RAPT, Takeda, UBS, and Pediapharma.

Igor Kaidashev has no conflict of interest to declare in relation to this work.

Iman Nasr has no conflict of interest to declare in relation to this work.

Isabel Ogueta Canales is or recently was a speaker and/or advisor for and/or has received personal fees and grants from L’Oréal, Faes‐Farma and ITF‐Labomed.

Ivan Cherrez‐Ojeda has no conflict of interest to declare in relation to this work.

Jean Bousquet has no conflict of interest to declare in relation to this work.

Jonathan A. Bernstein reports personal fees and grants from Sanofi Regeneron, AZ, Allakos, Celldex, Escient, Novartis, Genentech, Jasper Pharma and being AAAAI immediate past president, WAO BOD, AAAAI Foundation Chairperson, UCARE/ACARE centre, JTF practice parameters committee member.

Jonny Peter declares payments or honoraria for lectures from CSL Behring, Takeda, Novartis and Sanofi Regeneron, participation on a data safety monitoring Board or Adboard for Pharvaris and Astria. Further grants or contracts from Takeda, Kalvista, Astria and Pharvaris.

Jose Ignacio Larco Sousa has no conflict of interest to declare in relation to this work.

Kanokvalai Kulthanan declares payments or honoraria for lectures from Novartis, Menarini, and Sanofi Genzyme.

Karsten Weller declares grants or contracts from Novartis, Sanofi, and Noucor and consulting fees and adboard participation for Novartis.

Kiran Godse has no conflict of interest to declare in relation to this work.

Krzysztof Rutkowski has no conflict of interest to declare in relation to this work.

Lasma Lapina has no conflict of interest to declare in relation to this work.

Laurence Bouillet has consulted/served as speaker for, engaged in research and educational projects with or accepted travel grants from the following companies: BioCryst, CSL Behring, Takeda, Novartis, GSK, Blueprint, Kalvista, Pharvaris.

Luis Felipe Ensina declares consulting fees from Sanofi, honoraria for lectures from Sanofi, Novartis, and Celltrion and travel grants from Sanofi.

Margarida Gonçalo declares consulting fees from AbbVie, Almirall, Biocryst, Boehringer Pfizer, and Novartis and honoraria for lectures from Sanofi, AbbVie, and Pfizer. Further support for travel grants from Sanofi and Almirall.

Maria Staevska has no conflict of interest to declare in relation to this work.

Mariam Ali Yousuf Al‐Nesf has no conflict of interest to declare in relation to this work.

Markus Magerl is an advisor for MOXIE.

Martin Metz has no conflict of interest to declare in relation to this work.

Martijn van Doorn declares grants from Janssen, Almirall, Third Harmonic, and Escient, payments for lectures from Novartis, AbbVie, Leo pharma, Sanofi, Janssen, UCB, and BMS and travel grants from UCB.

Mary Anne Castor has no conflict of interest to declare in relation to this work.

Maryam Khoshkhui has no conflict of interest to declare in relation to this work. Outside of submitted work she was a speaker and/or advisor for and/or has received research funding from Abidi Pharma, Alhavi Pharma, AstraZeneca, Actover, Ofogh Tolid Darou pars, Kimia salamat nikan, CinnaGen, Sanofi, GlaxoSmithKline and Danon.

Michael Makris has no conflict of interest to declare in relation to this work.

Michihiro Hide declares consulting fees from Novartis, Sanofi, Taiho, and Teikoku Seiyaku, and payments or honoraria for lectures from Japan Tobacco, Kaken Pharmaceutical, Kyorin Pharmaceutical, Mitsubishi Tanabe Pharma, Meiji Seiyaku, Novartis, Sanofi/Regeneron, and TAIHO Pharmaceutical. Further travel grants from TAIHO Pharmaceutical.

Mohamad Abuzakouk has no conflict of interest to declare in relation to this work.

Mona Al‐Ahmad declares grants or contracts with Kuwait University, payments or honoraria for lectures from AstraZeneca, Novartis, GSK, and Sanofi.

Murat Türk is or recently was a speaker and/or advisor for AstraZeneca, Chiesi, GSK, Novartis, ROXALL, Vem İlaç.

Natasa Teovska Mitrevska has no conflict of interest to declare in relation to this work.

Niall Conlon declares grants or contracts with GSK and Pharming, payments or honoraria for lectures from Novartis and Takeda. Further travel grants from Novartis, Biocryst, and Pharming.

Nicole Nojarov has no conflict of interest to declare in relation to this work.

Pavel Kolkhir declares grants or contracts with Novartis and Sanofi, further consulting fees from BioCryst, Merus, Novartis, and ValenzaBio.

Philip Li has no conflict of interest to declare in relation to this work.

Ramzy Mohammed Ali has no conflict of interest to declare in relation to this work.

Rand Arnaout has no conflict of interest to declare in relation to this work.

Riccardo Asero declares payments of honoraria from GSK, Novartis, Jasper Therapeutics, and Sanofi.

Sabine Altrichter declares grants or contracts with AstraZeneca, and consulting fees from Allakos, ALK, BioCryst, Blueprint, Celltrion, CSL Behring, Galderma, Incyte, KalVista, Leo Pharma, Novartis, Otsuka, Pharvaris, Sanofi, Takeda, and ThermoFisher. Further payments or honoraria for lectures from Allakos, ALK, BioCryst, Blueprint, CSL Behring, Leo Pharma, Novartis, Pharvaris, Sanofi, Takeda, and ThermoFisher. In addition she received travel grants from BioCryst, Takeda, Sanofi, and UCB. Also she was part of Adboards for BioCryst, Takeda, Novartis, AstraZeneca, and Blueprint.

Simon Francis Thomsen Simon Francis Thomsen has received research support from AbbVie, Almirall, Janssen, LEO Pharma, Novartis, Sanofi and UCB, and has been a speaker/consultant for AbbVie, Almirall, Boehringer, CSL, Eli Lilly, Galderma, Incyte, Janssen, LEO Pharma, Novartis, Pfizer, Sanofi, Servier, Symphogen, UCB, and Union Therapeutics.

Young‐Min Ye declares payments or honoraria for lectures from Novartis, Celltrion, and Yuhan.

Zenon Brzoza declares payments or honoraria for lectures from Novartis, Berlin Chemie, Menarini, AstraZeneca, and Sanofi.

Zuotao Zhao is the speaker/advisor for and/or has received research funding from Novartis, Sanofi, Pfizer, Astellas, Galderma, Janssen, GSK, BAYER, LEO, MEDA Pharma and ALK Pharma.

Torsten Zuberbier declares grants or contracts from Novartis and Henkel and consulting fees from Bayer Health Care, Blueprint Medicine, Celldex, Celltrion, FAES, Novartis, and Henkel. Further payments for lectures from AstraZeneca, AbbVie, ALK, Almirall, Astellas, Bayer Healthcare, Bencard, Berlin Chemie, FAES, HAL, Leti, Meda, Menarini, Merck, MSD, Novartis, Pfizer, Sanofi, Stallergenes, Takeda, Teva, UCB, Henkel, Kryolan, and L’Oréal. He was part of adboards for Novartis, Celldex, and AbiVax.

Frank Siebenhaar declares grants or contracts with Alys/Granular Pharma, Blueprint Medicine, Cogent Biosciences, Celldex, and Telios Pharma, consulting fees from Blueprint Medicine, and Cogent Biosciences, payments or honoraria for lectures from Blueprint Medicine, Cogent Biosciences, and Novartis. He was part of adboards for Blueprint and Cogent.

Emek Kocatürk was recently a speaker/consultant for Menarini and Novartis and received funding from Almirall.

Sophia Neisinger has been an advisor/speaker for or received research funding from Novartis, Celltrion and/or Sanofi.

## Supporting information


**Figure S1:** Bars plot about the correlation between age and CRUSE app.

## Data Availability

The data that support the findings of this study are available from the corresponding author upon reasonable request.
