# Supplementary material for: Key Predictors of Adherence to a Mobile Health App for Managing Chronic Spontaneous Urticaria
Source: Clin Transl Allergy. 2025 Nov 15;15(11):e70110. doi: 10.1002/clt2.70110 (PMC12619656; doi:10.1002/clt2.70110)
Supplement: Supplementary file 1 — Figure S1: Bars plot about the correlation between age and CRUSE app. [file CLT2-15-e70110-s001.docx]

**Supplementary material**

**
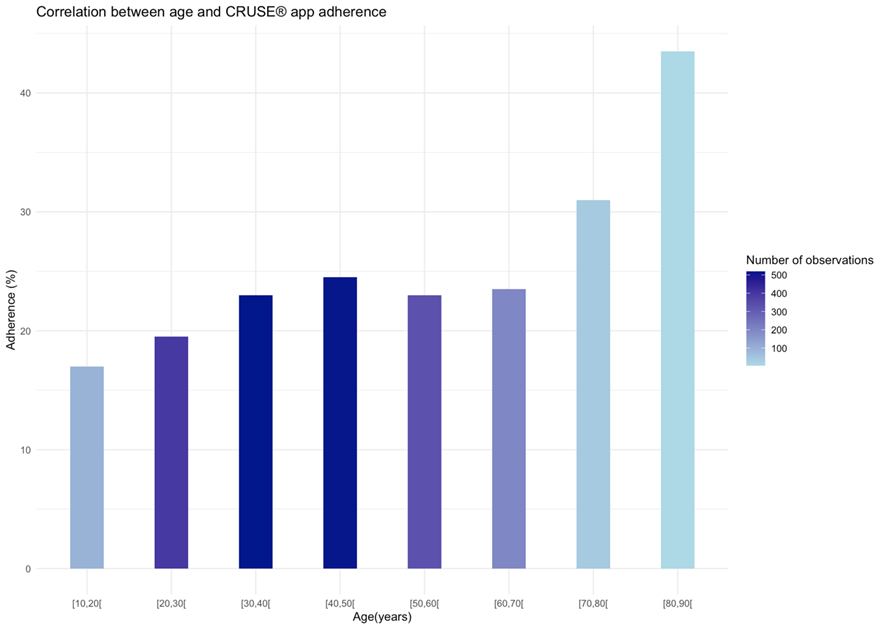
Supplementary Figure 1 – Bars plot about the correlation between age and CRUSE^®^ app**
